# Association between kidney function and the risk of cancer: Results from the China Health and Retirement longitudinal study (CHARLS)

**DOI:** 10.7150/jca.47175

**Published:** 2020-09-13

**Authors:** Lili Liu, Ming Zhu, Qinqin Meng, Yafeng Wang, Yaohui Zhao, Dawei Xie, Luxia Zhang, Ming-hui Zhao

**Affiliations:** 1 Renal Division, Department of Medicine, Peking University First Hospital; Institute of Nephrology, Peking University; Key Laboratory of Renal Disease, National Health and Family Planning Commission of the People's Republic of China; Key Laboratory of Chronic Kidney Disease Prevention and Treatment, Ministry of Education, Beijing, China.; 2 Zhongshan School of Medicine, Sun Yat-sen University, Guangzhou, Guangdong, China.; 3 Institute of Social Science Survey, Peking University, Beijing, China.; 4 National School of Development, Peking University, Beijing, China.; 5 Department of Biostatistics, Epidemiology, and Informatics, University of Pennsylvania Perelman School of Medicine, Philadelphia, U.S.A.; 6 National Institute of Health Data Science at Peking University, Beijing, China.; 7 Peking-Tsinghua Center for Life Sciences, Beijing, China.

**Keywords:** Kidney function decline, glomerular filtration rate, chronic kidney disease, cancer

## Abstract

**Objective:** Increased cancer risk after dialysis or transplantation has been recognized, but studies of cancer in pre-dialysis chronic kidney disease (CKD) are extremely limited. Therefore, we aim to investigate the risk of cancer in individuals with reduced kidney function.

**Methods:** This study was based on China Health and Retirement Longitudinal Study (CHARLS), a nationally representative population aged ≥ 45 years old. We included 11 508 (5364 male) individuals with measurement of serum creatinine and without history of cancer at baseline. Incident cancer cases were documented in the biennial questionnaire.

**Results:** The mean age was 58.7 ± 9.8 years. Participants with estimated glomerular filtration rate (eGFR) ≥ 90 ml/min/1.73m^2^, 60 to 89 ml/min/1.73m^2^, and eGFR < 60 ml/min/1.73m^2^ accounted for 62.9%, 33.7% and 3.4%, respectively. During 42 895 person-years' follow-up, 217 new cases of cancer were recorded. In participants with eGFR < 90 ml/min/1.73m^2^, cubic spline showed linear relationship between the risk of cancer and eGFR, while remained stable and no association in participants with eGFR > 90 ml/min/1.73m^2^. Compared to participants with eGFR ≥ 90 ml/min/1.73m^2^, those with eGFR < 60 ml/min/1.73m^2^ was associated with the increased risk of cancer in the fully adjusted model (hazard ratio 2.08; 95% confidence interval 1.22-3.53); and the risk for kidney and lung cancers was higher among those with eGFR < 60 ml/min/1.73m^2^.

**Conclusion:** Reduced kidney function is associated with a higher risk of cancer and should be integrated into risk-stratification of cancer screening and management.

## Introduction

The global burden of cancer is growing, particularly in low and middle-income countries 1. It is estimated that globally new cancer cases will be increased from 12.7 million in 2008 to 22.2 million in 2030 1. The need to implement effective strategies of risk identification and primary prevention is urgent.

Chronic kidney disease (CKD), with the prevalence of 10.4% in men (95% confidence interval [CI], 9.3% to 11.9%) and 11.8% in women (95% CI, 11.2% to 12.6%) worldwide 2, is associated with increased risk of multiple morbidities including end-stage kidney disease (ESKD) and cardiovascular disease. Furthermore, the increased risk of cancer has been reported among patients with ESKD on dialysis or receiving kidney transplantation 3-5. Recent epidemiologic studies revealed that earlier stages of CKD before ESKD were associated with increased risk of certain types of cancer, but the results were inconsistent 6-8. Besides, the association between estimated glomerular filtration rate (eGFR) and overall cancer was reported to be unrelated 7, 8, U-shaped 9, 10 or J-shaped association 11. In addition, cohort studies about their association among pre-dialysis CKD in representative populations are limited, especially from Asian population, among whom genetic background, behavior habits, as well as environmental settings are different from those of western countries.

Therefore, our study aims to investigate the association between kidney function and the risk of cancer by using data of China Health and Retirement Longitudinal Study (CHARLS), a nationally representative survey of general population aged 45 years or older in China.

## Methods

### Population

The current analysis was based on the CHARLS survey, which began in 2011 and was followed up every two years by the National School of Development of Peking University and with primary focus on health and retirement of the Chinese middle-aged and elder population. The detailed information about the survey has been described previously 12. Briefly, a nationally representative sample of 17 708 respondents aged 45 years or older was chosen in 150 randomly selected county-level units across the country with a probability-proportional-to-size method. The baseline survey was carried out between June 2011 and March 2012 with a response rate of 80.5%. Venous blood samples were collected from 11 847 individuals by medically trained staff from which serum creatinine was measured. The respondents were followed up twice in 2013 and 2015. Information on cancer is collected in every wave by means of self-reports or informants in case of death.

For the current analysis, 11 726 participants without history of cancer at baseline were included. Then, we excluded 5 participants with missing information of birthday, and 213 participants with missing baseline information of serum creatinine. Finally, 11 508 individuals were included for the analysis. The study was approved by the Biomedical Ethics Review Committee of Peking University in accordance with the ethical standards as laid down in the 1964 Declaration of Helsinki and its later amendments.

### Blood sample collection and analysis

Upon collection, all blood samples were immediately frozen at -20 °C and transported to the China Center of Disease Control in Beijing within 2 weeks where they were stored at -80^ ◦^C until assay at laboratory of Capital Medical University. Fasting blood glucose (FBG), total cholesterol (TC), high density lipoprotein cholesterol (HDL-C), low density lipoprotein cholesterol (LDL-C) and triglycerides (TG) were measured by enzymatic colormetric test. Hemoglobin A1c (HbA1c) was measured by boronate affinity High Performance Liquid Chromatography method. High-sensitivity C-reactive protein (hs-crp) was measured by immunoturbidimetric assay. All measurements were performed by trained medical personnel.

### Measurement of kidney function

Serum creatinine was measured by rate-blanked and compensated Jaffe creatinine method. The within and between-assay coefficients of variation of this method were < 1.6% and < 2.1%, respectively. Detection limits were 0.1~25 mg/dl. eGFR was calculated using the two-race CKD Epidemiological Collaboration equation 13. The level of eGFR was categorized into 3 groups: ≥ 90, 60 to 89, and < 60 ml/min/1.73m^2^.

### Definition of cancer

All participants were asked “Have you ever been diagnosed with cancer or malignant tumor (excluding minor skin cancers) by a doctor?” Participants with an affirmative answer were further asked “In which location of your body do you have cancer? Including the origins and metastasis of tumor (circle all that apply): 1) Brain; 2) Oral cavity; 3) Larynx; 4) Other pharynx; 5) Thyroid; 6) Lung;7) Breast; 8) Esophagus; 9) Stomach; 10) Liver; 11) Pancreas; 12) Kidney; 13) Prostate; 14) Testicle; 15) Ovary; 16) Cervix; 17) Endometrium; 18) Colon or rectum; 19) Bladder; 20) Skin; 21) Non-Hodgkin lymphoma; 22) Leukemia; 23) Other location”. Participants with affirmative answer were classified as having cancer.

Furthermore, participants who died during the study period and with cancer listed as the cause of death were also identified as having incident cancer.

### Other variables

The CHARLS baseline questionnaire includes information on demographics (e.g., age, sex), socio-economic factors (e.g., education and per capita expenditure [PCE]), lifestyle and health-related behaviors (e.g., smoking and drinking), and self-reported chronic illness (e.g., diabetes, hypertension). Blood pressure was measured three times with Omron HEM-7200 Monitor according to the protocol and the average of the three readings was used. The anthropometric measurements also were measured. Body mass index (BMI) was calculated as weight in kilograms divided by height in meters squared. BMI was classified as underweight (< 18.5 kg/m^2^), normal (18.5-23.9 kg/m^2^), overweight (24.0-27.9 kg/m^2^) and obesity (≥ 28 kg/m^2^) according to the guideline from the Ministry of Health of the People's Republic of China. Hypertension was defined as SBP ≥ 140 mmHg, and/or DBP ≥ 90 mmHg, and/or self-reported hypertension. Diabetes was defined as FBG ≥ 126 mg/dl, and/or HbA1c ≥ 6.5%, and/or self-reported history of diabetes. Dyslipidemia was defined as the presence of at least one of following: serum TC ≥ 200 mg/dl, TG ≥ 150 mg/dl, LDL-C ≥ 130 mg/dl, HDL-C < 40 mg/dl. Micro-inflammatory state was defined as serum hs-crp ≥ 3 mg/dl. A strict quality control of data recording and checking was conducted to ensure data reliability.

### Statistical analyses

The baseline characteristics of participants were described and compared according to the three categories of baseline eGFR (≥ 90, 60 to 89, and < 60 ml/min/1.73m^2^). Continuous variables were presented as mean (standard deviation, SD) for normal distribution and as median (interquartile range, IQR) for skewed distribution. Categorical variables were presented as number (percentage). Baseline characteristics of all participants were compared using the analysis of variance or Kruskal-Wallis test for continuous variables depending on data distribution and the χ^2^ test for categorical variables.

Cox proportional hazard model was used to examine the association between eGFR groups (≥ 90 [reference], 60 to 89, and < 60 ml/min/1.73m^2^) and the risk of cancer. Participants who did not develop cancer during follow up and died of causes unrelated to cancer and lost to follow-up were censored. Before running Cox regression, we conducted multiple imputation (5 datasets) for the cancer incidence time. The final estimate of the event rate would be the average of the event rates from the 5 datasets. If a patient was censored, then we did not do any imputation. Incidence rates of cancer were expressed as per 100,000 person-years. In Cox regression, the unadjusted hazard ratios (HRs) with 95% confidence intervals (CIs) were reported. Then, age, sex and smoking (never vs. past vs. current) were included as potential confounders. In fully adjusted model, household registration status (rural vs. urban), educational level (above vs. below high school) and PCE (tertile), drinking habits (never vs. past vs. current), BMI (categories), diabetes, hypertension, dyslipidemia, as well as micro-inflammatory state were also included. PH assumption was tested by Schoenfeld residuals and it was held for all the variables in the Cox model (*P* = 0.96). eGFR was also analyzed as a continuous variable.

We further evaluated dose-response relationship between eGFR and risk of cancer by using restricted cubic spline with knots of 20th, 40th, 60th and 80th percentiles and eGFR = 90 ml/min/1.73m^2^ as the reference. The same potential confounding factors in fully adjusted model were adjusted in restricted cubic spline models.

Since cancer associated with abnormalities and treatment could lead to kidney injury, we further conducted a sensitivity analysis by excluding participants who developed cancer in the first follow-up time (2013) to avoid reverse causality.

For the site-specific cancer, the number was listed and compared between different eGFR groups, and Kaplan-Meier curves were plotted.

Statistical analyses were conducted by using Stata/MP version 14.0 for Windows (Stata Corp., College Station, Texas, USA), with *α* < 0.05 in two-side test considered statistically significant.

## Results

### Baseline characteristics of study population

The mean age of the 11 508 participants was 58.7 ± 9.8 years and 5364 (46.6%) participants were male. Characteristics of the participants at baseline were described in overall and compared according to eGFR groups (**Table 1**). The prevalence of obesity, hypertension, diabetes and dyslipidemia was 4.8%, 40.8%, 16.3% and 62.7%, respectively. Participants with lower eGFR were older and had higher prevalence of obesity, hypertension, diabetes and dyslipidemia.

### Kidney function

A total of 3880 (33.7%) individuals had eGFR between 60 and 89 ml/min/1.73m^2^, with the mean eGFR of 79.2 (SD, 8.0) ml/min/1.73m^2^. A smaller number of 393 (3.4%) individuals had eGFR less than 60 ml/min/1.73m^2^, with the mean eGFR of 50.2 (SD, 9.6) ml/min/1.73m^2^. Only 4 individuals had eGFR less than 15 ml/min/1.73m^2^.

### Cumulative incidence of cancer

Median follow-up for the cohort was 4.0 years, with a total of 42 895 person-years of follow-up. During follow-up, 217 new cases of cancer (excluding minor skin cancers) were recorded (105 died during the follow-up). Among those cases, 117, 79 and 21 were identified in the group of eGFR ≥ 90, 60 to 89, and < 60 ml/min/1.73m^2^, respectively. The incidence rates of cancer were 506 per 100 000 person-years in overall, and 431, 550 and 1515 per 100 000 person-years in groups of eGFR ≥ 90, 60 to 89 and < 60 ml/min/1.73m^2^, respectively (**Table 2**).

### Association between kidney function and the risk of cancer

The HRs and 95% CIs for the association between different groups of eGFR and the risk of cancer were shown in **Table 2.** Compared to those with eGFR ≥ 90 ml/min/1.73m^2^, the fully adjusted HR and 95% CI of those with eGFR < 60 ml/min/1.73m^2^ was 2.08 (95% CI: 1.22 to 3.53). If eGFR was analyzed as a continuous variable, the fully adjusted HR for per 10 ml/min/1.73m^2^ decrease was 1.09 (95% CI: 0.99, 1.20). The association did not change substantially in the sensitivity analysis (**Table 3**).

### The dose-response relationship between eGFR and risk of cancer

**Figure 1:**
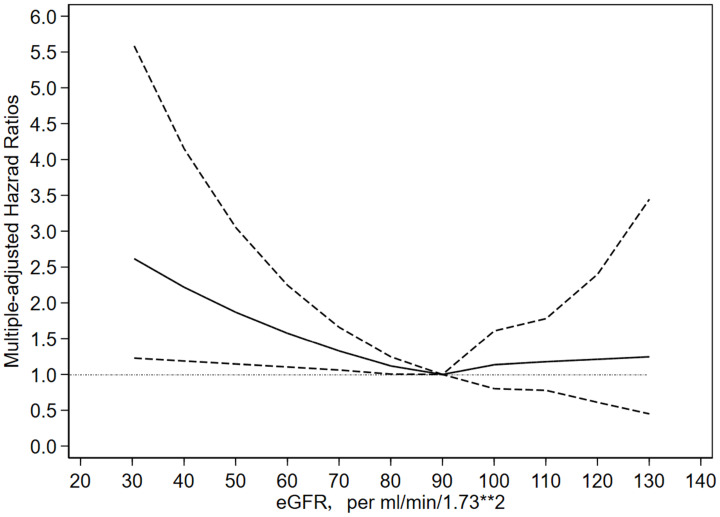
** The relationship between eGFR and risk of cancer using adjusted restricted cubic spline model.** Note: The reference value for each hazard ratio is the eGFR = 90 ml/min/1.73m^2^. The solid line represented estimated hazard ratio and the dotted line indicated 95% confidence interval.

As shown in **Figure 1**, the lowest risk of cancer seems to be eGFR of about 90 ml/min/1.73m^2^, while gradually, linearly and significantly increase when eGFR is less than 90 ml/min/1.73m^2^, and remained stable and no association when eGFR is greater than 90 ml/min/1.73m^2^.

### Site-specific cancer

As shown in **Table 4**, the number of cancer at specific sites was small and the top 5 common sites of cancer located in lung (n = 45), liver (n = 33), esophagus (n = 24), stomach (n = 19) and cervix (n = 17). The incidence rates of site-specific cancer between different eGFR groups have statistical difference in lung (*P* = 0.001), liver (*P* = 0.02), kidney (*P* = 0.007), colon or rectum (*P* = 0.004), bladder (*P* = 0.05), skin (*P* = 0.05) and other location (*P* = 0.001).

**Figure 2:**
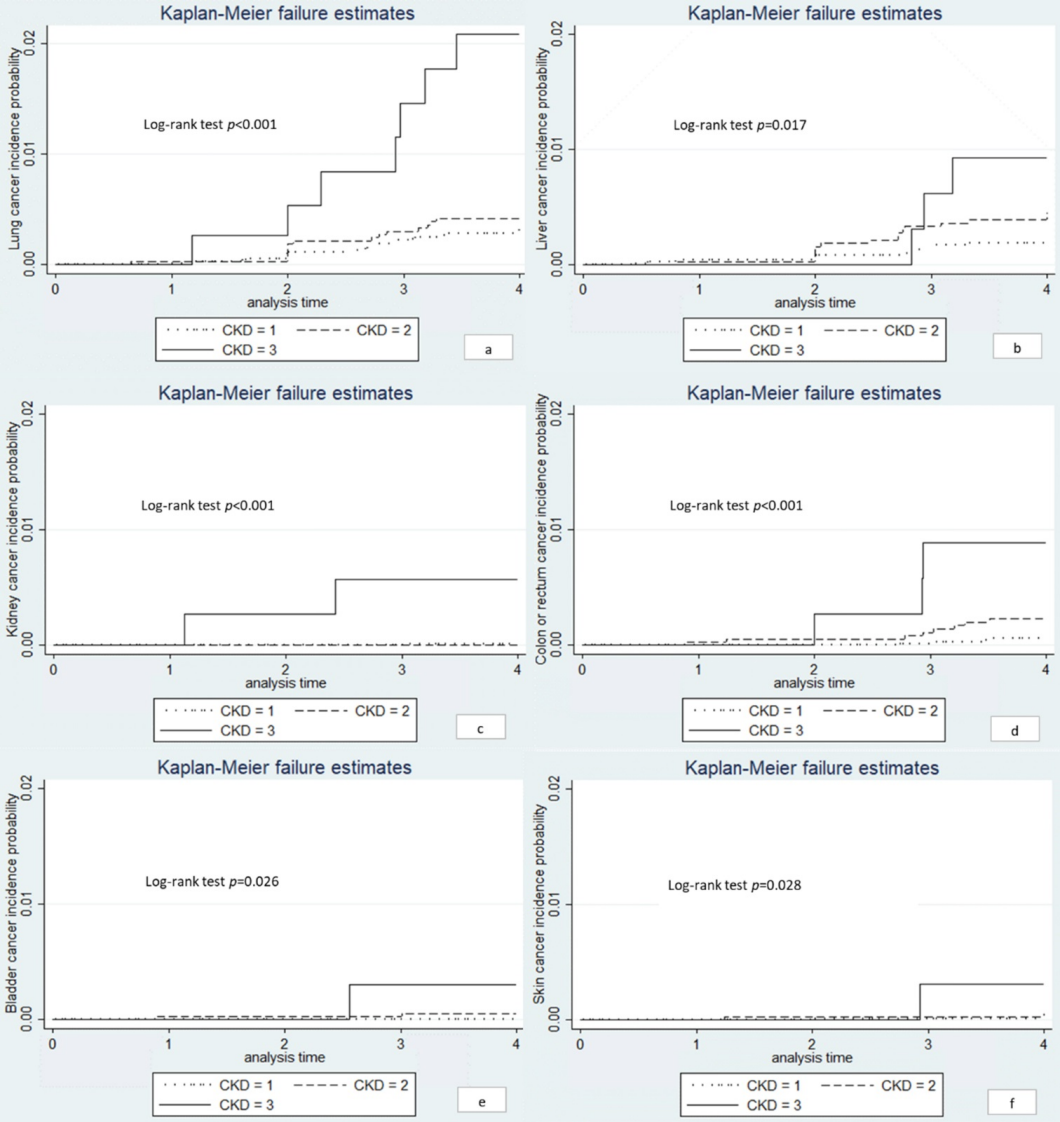
** Kaplan-Meier curves for site-specific cancers.** Note: *^a^* lung cancer; *^b^* liver cancer; *^c^* kidney cancer; *^d^* colon or rectum cancer; *^e^* bladder cancer; *^f^* skin cancer. CKD1: eGFR ≥ 90 ml/min/1.73m^2^; CKD2: eGFR 60-90 ml/min/1.73m^2^; CKD3: eGFR < 60 ml/min/1.73m^2^.

We conducted the Kaplan-Meier curves for the site-specific cancer located in lung, liver, kidney, colon or rectum and skin (**Figure 2**). Significant differences were observed between the three eGFR groups (log-rank test, *P* < 0.05). The univariate Cox regression showed that the risk of lung cancer, kidney cancer, and colon or rectum cancer were significantly higher among individuals with eGFR < 60 ml/min/1.73m^2^ compared to those with eGFR > 90 ml/min/1.73m^2^ (**Table 5**). In age-sex adjusted Cox regression, results remained only in lung and kidney cancer (**Table 5**). However, the association between eGFR < 60 ml/min/1.73m^2^ and lung cancer was eliminated after further controlling for smoking (HR: 2.69; 95% CI: 0.98-7.38, *P* = 0.054). We did not have sufficient statistical power to conduct further multivariate analysis.

## Discussion

Based on the prospective cohort study that included a large representative sample of the general population, we found a two-fold increased risk of overall cancer in participants with reduced kidney function, independent of many other known risk factors. Our results revealed that reduced kidney function should be considered as an independent risk factor of cancer. Meanwhile, intensive monitoring of cancer is necessary for patients with CKD.

Increased risk of cancer has been well documented among patients on dialysis or receiving kidney transplantation. A high burden of cancer in the dialysis population was reported by the annual data report of United States Renal Data System (USRDS), with an incidence rate of site-specific cancer from 3923 to 3860 cases per 100 000 person-years 5. An earlier study including patients from US, Europe, Australia, and New Zealand showed the risk of virus-related cancers being higher in patients on dialysis than in the general population as well 14. Furthermore, kidney transplantation is reported to be associated with a marked increase in cancer risk at a wide variety of sites 4.

However, studies based on the representatively general population investigating the association between pre-dialysis CKD and the risk of cancer are relatively limited. The Blue Mountains Eye Study, which is a cohort study of 3654 residents aged 49 to 97 years in Australia, reported an excess risk of overall cancer (especially for lung and urinary tract cancers) in men with eGFR < 55 ml/min/1.73m^2^. For every 10 ml/min/1.73m^2^ decline in eGFR, the risk of cancer increased by 29% independent of age and smoking 6. Studies based on cancer registry or claims data described similar results 8, 10. However, there are also meta-analysis or study including patients with diabetes and CKD revealed conflicted results 15, 16. The possible reasons of inconsistent results include the heterogeneity of study populations, inclusion bias caused by non-representative population, as well as relatively small sample size 6, and short follow-up period 15, 16.

In addition, the evidence of the association between CKD and the risk of cancer mostly come from western countries. Studies from Asian countries are extremely limited, where the burden of cancer is also striking. It is estimated that nearly one-half of the cancer cases and over one-half of the cancer deaths in the world occur in Asia 17. The Korean Heart Study including 242 583 adults reported a “J-shaped” relationship between eGFR and incidence of any cancer, with the lowest risk at 45 to 59 ml/min/1.73m^2^ 11. However, the cubic spline in our study showed that the lowest risk of cancer is around eGFR = 90 ml/min/1.73m^2^. The analysis based on data of the Stockholm Creatinine project including 719 033 Swedes with 5 years' follow-up and the MJ Cohort study including 405 878 Taiwanese with 8.7 years' follow-up partly supported our results 9, 10. Whereas, unlike “U-shaped” relationship, our finding indicated that there are no relationship between eGFR > 90 ml/min/1.73m^2^ and the risk of cancer, the possible reason might be a relatively small sample in our study or inclusion bias in previous studies. Overall, our study further expanded previous observation to mainland Chinese population based on a nationally representative sample with a strict quality-control process.

The association between reduced kidney function seems to be site specific for lung and kidney cancers. This finding was consistent with the Blue Mountains Eye Study which including 3654 people aged older than 49 years 6. The reason for the eliminated association between reduced kidney function and lung cancer after adjusted for smoking maybe due to the small number of lung cancer cases. And we did not have the sufficient power to conduct further conclusive analyses.

The underlying mechanisms between the association with kidney function and the risk of cancer remains unclear, and there are several possible explanations. First, compared with persons of eGFR ≥ 90 ml/min/1.73m^2^, those of eGFR < 60 ml/min/1.73m^2^ tended to have more risk factors of cancer, such as older age, smoking, obesity and diabetes 18. Second, CKD and cancer have common risk factors, such as virus and herbal medicines including aristolochine, which have both nephrotoxic effects and carcinogenic activity 19, 20. Third, the retention of uremic toxins could result in immunosuppression, which contribute to the development of cancer, and further accentuate mitogenesis and proliferation in malignant tumors. Furthermore, reduced kidney function could lead to micro-inflammation and oxidative stress 21, which may playing a role in transformation of normal cells to tumor cells 22. Finally, the treatment of CKD such as glucocorticoid and immunosuppressant may have effect on development of cancer 23. Further studies are required to explore the potential mechanisms of the association between kidney function and the risk of cancer.

Our study has several strengths. First, our study is the first one in Asia that is based on a national representative population aged over 45 years old, with a good generalizability among those population. Second, a strict quality control of data recording and checking was conducted to ensure data reliability. Finally, multiple potential confounders were included in the analyses, and a sensitivity analysis was conducted to avoid reverse causality. Meanwhile, our study also has notable limitations. First, relatively short period of follow-up limited our ability to perform stratified analyzed based on sites of cancer. Second, the incident cancer cases were identified by self-reporting, while a prospective cohort study in comparison with data from State Cancer Registries have reported a reasonably high validity for self-reported cancer with the sensitivity up to 0.93 and the calculated specificity up to 0.98, as well as the overall positive predictive values of 0.75 24. Furthermore, the age-specific cancer incidence in our study was comparable to the report from the National Central Cancer Registry in China 25. Finally, previous study revealed that the level of albuminuria is independently associated with the risk cancer and is therefore a potential confounder in the eGFR-cancer link 10. However, we do not have results of information of albuminuria in our study and the possibility of residual confounders exists.

## Conclusion

In conclusion, our study showed that reduced kidney function is associated with an increased risk of overall cancer and therefore should be integrated into the risk-stratification of cancer management.

## Figures and Tables

**Table 1:** Characteristics of the participants at baseline in 2011 by eGFR (ml/min/1.73m^2^) groups

Characteristics	Total (n = 11 508)	eGFR ≥ 90 (n=7235)	eGFR 60 to 89 (n = 3880)	eGFR < 60 (n = 393)	*P* value
Age, mean (SD)	58.7 (9.8)	55.3 (7.8)	63.8 (9.9)	70.7 (9.7)	<0.001
Male, N (%)	5364 (46.6)	3282 (45.4)	1888 (48.7)	194 (49.6)	0.002
Rural, N (%)	9370 (81.5)	6077 (84.0)	3007 (77.6)	286 (72.8)	<0.001
Above high school, N (%)	1229 (10.7)	826 (11.4)	374 (9.7)	29 (7.4)	0.002
PCE, median (IQR)	6647.4 (4073.9, 11207.7)	6574.4 (4104.5, 11081.2)	6765.1 (40034.0, 11316.8)	7335.2 (3972.6, 10804.8)	0.63
**Smoking, N (%)**					
Never	7005 (62.3)	4487 (63.4)	2283 (60.4)	235 (61.4)	<0.001
Past	953 (8.5)	504 (7.1)	395 (10.5)	54 (14.1)	
current	3280 (29.2)	2084 (29.5)	1102 (29.2)	94 (24.5)	
**Drinking, N (%)**					
Never	6762 (59.0)	4248 (59.0)	2262 (58.6)	252 (64.6)	<0.001
Past	938 (8.2)	484 (6.7)	396 (10.3)	58 (14.9)	
current	3756 (32.8)	2474 (34.3)	1202 (31.1)	80 (20.5)	
SBP, mean (SD)	129.6(21.6)	127.1 (20.5)	133.1 (22.6)	141.2 (23.5)	<0.001
DBP, mean (SD)	75.3 (12.2)	75.2 (12.2)	75.3 (12.2)	77.3 (13.4)	0.01
**BMI, N (%)**					
Underweight	656 (6.8)	347 (5.7)	279 (8.5)	30 (9.3)	<0.001
Normal	5978 (61.9)	3787 (62.7)	2000 (60.8)	191 (59.3)	
Overweight	2553 (26.5)	1616 (26.7)	856 (26.0)	81 (25.2)	
Obesity	465 (4.8)	291 (4.8)	154 (4.7)	20 (6.2)	
TC, mean (SD)	192.9 (38.6)	190.3 (38.0)	197.0 (39.1)	200.4 (41.2)	<0.001
HDL-C, mean (SD)	50.9 (15.3)	51.2 (15.4)	50.4 (15.2)	50.3 (15.3)	0.03
LDL-C, mean (SD)	116.1 (34.9)	114.1 (34.2)	119.4 (35.5)	120.0 (37.5)	<0.001
TG, median (IQR)	106.2 (75.2, 155.8)	102.7 (73.5, 151.3)	110.6 (78.8, 162.4)	117.7 (83.2, 176.1)	<0.001
Serum creatinine, median (IQR)	0.8 (0.7, 0.9)	0.7 (0.6, 0.8)	0.9 (0.8, 1.0)	1.2 (1.1, 1.4)	<0.001
eGFR, mean (SD)	92.3 (15.3)	101.6 (7.6)	79.2 (8.0)	50.2 (9.6)	<0.001
FBG, median (IQR)	102.4 (94.3, 113.6)	101.9 (94.0, 112.3)	103.1 (95.0, 115.0)	108.0 (97.5,122.1)	<0.0001
HbA1c, median (IQR)	5.1 (4.9, 5.4)	5.1 (4.9, 5.4)	5.1 (4.9, 5.5)	5.2 (4.9, 5.5)	0.002
Hs-CRP, median (IQR)	1.0 (0.6, 2.2)	0.9 (0.5, 2.0)	1.2 (0.6, 2.4)	1.8 (1.0, 4.4)	<0.001
Micro-inflammatory state, N (%)	2067 (18.0)	1162 (16.1)	771 (19.9)	134 (34.1)	<0.001
Hypertension, N (%)	3948 (40.8)	2136 (35.3)	1602 (48.6)	210 (64.4)	<0.001
Diabetes, N (%)	1642 (16.3)	955 (15.0)	591 (17.3)	96 (28.5)	<0.001
Dyslipidemia, N (%)	7217 (62.7)	4368 (60.4)	2581 (66.5)	268 (68.2)	<0.001

Note:^*a*^ Calculated with CKD-EPI equation based on serum creatinine (ml/min/1.73m^2^). Abbreviation: PCE: per capita expenditure; SBP: systolic blood pressure; DBP: diastolic blood pressure; BMI: body mass index; TC: total cholesterol; HDL-C: high density lipoprotein cholesterol; LDL-C: low density lipoprotein cholesterol; TG: triglyceride; eGFR: estimated glomerular filtration rate; FBG: fasting blood glucose; HbA1c: hemoglobin A1c; Hs-crp: high sensitivity C reactive protein.

**Table 2:** Hazard ratios (HRs) and 95% confidence intervals (CIs) for risk of cancer in different eGFR groups

eGFR (ml/min/1.73m^2^)	N (%)	No. of case	Incidence rate	HR (95% CI) *^a^*	*P*	HR (95% CI) *^b^*	*P*	HR (95% CI) *^c^*	*P*	HR (95% CI) *^d^*	*P*
≥ 90	7235 (62.9%)	117	431	1 [reference]	-	1 [reference]	-	1 [reference]	-	1 [reference]	-
60 to 89	3880 (33.7%)	79	550	1.28 (0.96, 1.70)	0.09	1.05 (0.76, 1.44)	0.78	1.01 (0.73, 1.39)	0.96	1.01 (0.73, 1.39)	0.96
< 60	393 (3.4%)	21	1515	3.57 (2.24, 5.68)	<0.001	2.48 (1.47, 4.18)	0.001	2.24 (1.32, 3.80)	0.003	2.08 (1.22, 3.53)	0.007
*Per* 10 units decrease	11 508	217	506	1.20 (1.11, 1.30)	<0.001	1.12 (1.02, 1.23)	0.02	1.10 (1.00, 1.21)	0.06	1.09 (0.99,1.20)	0.09

Note: Incidence rates were expressed as per 100 000 person-years. *^a^* Unadjusted; *^b^* Adjusted for age, sex and smoking; *^c^* Adjusted for age, sex, smoking, education, PCE, BMI, drinking, diabetes, hypertension, dyslipidemia;*^d^* Adjusted for all above and micro-inflammation.

**Table 3:** Sensitivity analysis of the association between different eGFR groups and risk of cancer

eGFR (ml/min/1.73m^2^)	N	No. of cases	HR (95% CI)*^a^*	*P*	HR (95% CI)*^b^*	*P*	HR (95% CI)*^c^*	*P*	HR (95% CI)*^d^*	*P*
≥ 90	7179	61	1 [reference]	-	1 [reference]	-	1 [reference]	-	1 [reference]	
60 to 89	3846	45	1.41 (0.96, 2.07)	0.08	1.03 (0.66, 1.57)	0.90	1.00 (0.65, 1.54)	0.92	1.00 (0.65, 1.54)	0.93
< 60	386	14	4.68 (2.61, 8.37)	<0.001	2.61 (1.34, 5.09)	0.01	2.41 (1.22, 4.75)	0.01	2.27 (1.15, 4.47)	0.02
*Per* 10 units decrease	11 411	120	1.24 (1.12, 1.38)	<0.001	1.10 (0.97, 1.26)	0.15	1.09 (0.95, 1.24)	0.21	1.07 (0.94, 1.23)	0.30

Note: *^a^* Unadjusted; *^b^* Adjusted for age, sex and smoking; *^c^* Adjusted for age, sex, smoking, education, PCE, BMI, drinking, diabetes, hypertension, dyslipidemia;*^d^* Adjusted for all above and micro-inflammation.

**Table 4:** Number of site-specific cancer and comparison between different eGFR groups

Site-specific cancer	Total number	Number of cancer according to eGFR (ml/min/1.73m^2^)			*P*
		eGFR ≥ 90	eGFR 60 to 89	eGFR < 60	
brain	5	4	1	0	0.72
Oral cavity	2	1	1	0	1.00
Larynx	7	5	1	1	0.23
Other pharynx	5	3	1	1	0.20
Thyroid	7	6	1	0	0.56
Lung	43	21	15	7	0.001
Breast	12	9	3	0	0.71
Esophagus	24	14	10	0	0.61
Stomach	19	12	6	1	0.66
Liver	33	14	16	3	0.02
Pancreas	7	4	3	0	0.76
Kidney	4	1	1	2	0.007
Prostate	3	2	0	1	0.09
Testicle	0	0	0	0	-
Ovary	4	4	0	0	0.40
Cervix	14	11	3	0	0.63
Endometrium	17	15	2	0	0.13
Colon or rectum	16	5	8	3	0.004
Bladder	4	1	2	1	0.05
Skin	4	1	2	1	0.05
Non-Hodgkin lymphoma	4	3	1	0	1.00
Leukemia	0	0	0	0	-
Other location	35	14	15	6	0.001

Note: Fisher's exact tests were used due to the expected frequency <1.

**Table 5:** Hazard ratios (HRs) and 95% confidence intervals (CIs) for reduced kidney function and risk of site-specific cancer

Site-specific cancer	Unadjusted		Adjusted	
	HR (95% CI)	*P*	HR (95% CI)	*P*
Lung	4.88 (1.99-11.97)	0.001	3.26 (1.30-8.20)	0.01
Lung *^a^*	-	-	2.69 (0.98-7.38)	0.05
Liver	1.06 (0.96-1.19)	0.25	-	-
Kidney	21.1 (1.91-232.6)	0.01	27.57 (2.23-341.27)	0.01
Colon or rectum	3.98 (1.06-15.00)	0.04	3.02 (0.77-11.94)	0.11
Bladder	5.27 (0.48-58.13)	0.18	-	-
Skin	5.21 (0.47-57.42)	0.18	-	-
Other location	4.19 (1.63-10.80)	0.003	3.81 (1.43-10.17)	0.01

Note: Adjusted for age and sex. Lung *^a^* was adjusted for age, sex and smoking.

## References

[1] Vineis P, Wild CP (2014). Global cancer patterns: causes and prevention. Lancet.

[2] Mills KT, Xu Y, Zhang W, Bundy JD, Chen CS, Kelly TN (2015). A systematic analysis of worldwide population-based data on the global burden of chronic kidney disease in 2010. Kidney Int.

[3] Birkeland SA, Lokkegaard H, Storm HH (2000). Cancer risk in patients on dialysis and after renal transplantation. Lancet.

[4] Vajdic CM, McDonald SP, McCredie MR, van Leeuwen MT, Stewart JH, Law M (2006). Cancer incidence before and after kidney transplantation. JAMA.

[5] Butler AM, Olshan AF, Kshirsagar AV, Edwards JK, Nielsen ME, Wheeler SB (2015). Cancer incidence among US Medicare ESRD patients receiving hemodialysis, 1996-2009. Am J Kidney Dis.

[6] Wong G, Hayen A, Chapman JR, Webster AC, Wang JJ, Mitchell P (2009). Association of CKD and cancer risk in older people. J Am Soc Nephrol.

[7] Christensson A, Savage C, Sjoberg DD, Cronin AM, O'Brien MF, Lowrance W (2013). Association of cancer with moderately impaired renal function at baseline in a large, representative, population-based cohort followed for up to 30 years. Int J Cancer.

[8] Lowrance WT, Ordonez J, Udaltsova N, Russo P, Go AS (2014). CKD and the risk of incident cancer. J Am Soc Nephrol.

[9] Xu H, Matsushita K, Su G, Trevisan M, Arnlov J, Barany P (2019). Estimated Glomerular Filtration Rate and the Risk of Cancer. Clin J Am Soc Nephrol.

[10] Tu H, Wen CP, Tsai SP, Chow WH, Wen C, Ye Y (2018). Cancer risk associated with chronic diseases and disease markers: prospective cohort study. BMJ.

[11] Mok Y, Matsushita K, Ballew SH, Sang Y, Jung KJ, Lee S (2017). Kidney Function, Proteinuria, and Cancer Incidence: The Korean Heart Study. Am J Kidney Dis.

[12] Zhao Y, Hu Y, Smith JP, Strauss J, Yang G (2014). Cohort profile: the China Health and Retirement Longitudinal Study (CHARLS). Int J Epidemiol.

[13] Kong X, Ma Y, Chen J, Luo Q, Yu X, Li Y (2013). Evaluation of the Chronic Kidney Disease Epidemiology Collaboration equation for estimating glomerular filtration rate in the Chinese population. Nephrol Dial Transplant.

[14] Maisonneuve P, Agodoa L, Gellert R, Stewart JH, Buccianti G, Lowenfels AB (1999). Cancer in patients on dialysis for end-stage renal disease: an international collaborative study. Lancet.

[15] Wong G, Zoungas S, Lo S, Chalmers J, Cass A, Neal B (2012). The risk of cancer in people with diabetes and chronic kidney disease. Nephrol Dial Transplant.

[16] Wong G, Staplin N, Emberson J, Baigent C, Turner R, Chalmers J (2016). Chronic kidney disease and the risk of cancer: an individual patient data meta-analysis of 32,057 participants from six prospective studies. BMC Cancer.

[17] Bray F, Ferlay J, Soerjomataram I, Siegel RL, Torre LA, Jemal A (2018). Global cancer statistics 2018: GLOBOCAN estimates of incidence and mortality worldwide for 36 cancers in 185 countries. CA Cancer J Clin.

[18] Giovannucci E, Harlan DM, Archer MC, Bergenstal RM, Gapstur SM, Habel LA (2010). Diabetes and cancer: a consensus report. Diabetes Care.

[19] Lin YS, Yeh CC, Lin YC, Su CT, Sung FC, Chang SN (2017). Kidney Cancer Linked to Chronic Hepatitis in the Asia-Pacific: A Population-Based Analysis. Am J Nephrol.

[20] Arlt VM, Stiborova M, Schmeiser HH (2002). Aristolochic acid as a probable human cancer hazard in herbal remedies: a review. Mutagenesis.

[21] Shlipak MG, Fried LF, Crump C, Bleyer AJ, Manolio TA, Tracy RP (2003). Elevations of inflammatory and procoagulant biomarkers in elderly persons with renal insufficiency. Circulation.

[22] Touvier M, Fezeu L, Ahluwalia N, Julia C, Charnaux N, Sutton A (2013). Association between prediagnostic biomarkers of inflammation and endothelial function and cancer risk: a nested case-control study. Am J Epidemiol.

[23] Engberg H, Wehberg S, Bistrup C, Heaf J, Sorensen SS, Thiesson HC (2016). Cancer risk and mortality after kidney transplantation: a population-based study on differences between Danish centres using standard immunosuppression with and without glucocorticoids. Nephrol Dial Transplant.

[24] Bergmann MM, Calle EE, Mervis CA, Miracle-McMahill HL, Thun MJ, Heath CW (1998). Validity of self-reported cancers in a prospective cohort study in comparison with data from state cancer registries. Am J Epidemiol.

[25] Chen W, Zheng R, Zuo T, Zeng H, Zhang S, He J (2016). National cancer incidence and mortality in China, 2012. Chin J Cancer Res.

